# A Study of Carry-Over and Histopathological Effects after Chronic Dietary Intake of Citrinin in Pigs, Broiler Chickens and Laying Hens

**DOI:** 10.3390/toxins12110719

**Published:** 2020-11-16

**Authors:** Celine Meerpoel, Arnau Vidal, Emmanuel K. Tangni, Bart Huybrechts, Liesbeth Couck, Riet De Rycke, Lobke De Bels, Sarah De Saeger, Wim Van den Broeck, Mathias Devreese, Siska Croubels

**Affiliations:** 1 Department of Bioanalysis, Centre of Excellence in Mycotoxicology and Public Health, Faculty of Pharmaceutical Sciences, Ghent University, Ottergemsesteenweg 460, 9000 Ghent, Belgium; Celine.Meerpoel@UGent.be (C.M.); Arnau.Vidalcorominas@UGent.be (A.V.); Sarah.Desaeger@UGent.be (S.D.S.); 2 Department of Pharmacology, Toxicology and Biochemistry, Faculty of Veterinary Medicine, Ghent University, Salisburylaan 133, 9820 Merelbeke, Belgium; Mathias.Devreese@UGent.be; 3 Sciensano, Chemical and Physical Health Risks, Organic Contaminants and Additives, Leuvensesteenweg 17, 3080 Tervuren, Belgium; Emmanuel.Tangni@Sciensano.be (E.K.T.); Bart.Huybrechts@Sciensano.be (B.H.); 4 Department of Morphology, Faculty of Veterinary Medicine, Ghent University, Salisburylaan 133, 9820 Merelbeke, Belgium; Liesbeth.Couck@UGent.be (L.C.); Lobke.Debels@UGent.be (L.D.B.); Wim.Vandenbroeck@ugent.be (W.V.d.B.); 5 Department of Biomedical Molecular Biology, Ghent University, Technologiepark Zwijnaarde 71, VIB Center for Inflammation Research, VIB Center for Inflammation Research, 9052 Ghent, Belgium; riet.derycke@ugent.be; 6 Ghent University Expertise Centre for Transmission Electron Microscopy, VIB BioImaging Core, Technologiepark Zwijnaarde 71, 9052 Ghent, Belgium; 7 Department of Biotechnology and Food Technology, Faculty of Science, Doornfontein Campus, University of Johannesburg, Gauteng 2028, South Africa

**Keywords:** citrinin, broiler chickens, laying hens, pigs, chronic dietary intake, carry-over, depletion, toxicity

## Abstract

Citrinin (CIT) is a polyketide mycotoxin occurring in a variety of food and feedstuff, among which cereal grains are the most important contaminated source. Pigs and poultry are important livestock animals frequently exposed to mycotoxins, including CIT. Concerns are rising related to the toxic, and especially the potential nephrotoxic, properties of CIT. The purpose of this study was to clarify the histopathological effects on kidneys, liver, jejunum and duodenum of pigs, broiler chickens and laying hens receiving CIT contaminated feed. During 3 weeks, pigs (n = 16) were exposed to feed containing 1 mg CIT/kg feed or to control feed (n = 4), while 2 groups of broiler chickens and laying hens (n = 8 per group) received 0.1 mg CIT/kg feed (lower dose group) and 3 or 3.5 mg CIT/kg feed (higher dose group), respectively, or control feed (n = 4). CIT concentrations were quantified in plasma, kidneys, liver, muscle and eggs using a validated ultra-high performance liquid chromatography-tandem mass spectrometry (UHPLC-MS/MS) method. Kidneys, liver, duodenum and jejunum were evaluated histologically using light microscopy, while the kidneys were further examined using transmission electron microscopy (TEM). Histopathology did not reveal major abnormalities at the given contamination levels. However, a significant increase of swollen and degenerated mitochondria in renal cortical cells from all test groups were observed (*p* < 0.05). These observations could be related to oxidative stress, which is the major mechanism of CIT toxicity. Residues of CIT were detected in all collected tissues, except for muscle and egg white from layers in the lowest dose group, and egg white from layers in the highest dose group. CIT concentrations in plasma ranged between 0.1 (laying hens in lower dose group) and 20.8 ng/mL (pigs). In tissues, CIT concentrations ranged from 0.6 (muscle) to 20.3 µg/kg (liver) in pigs, while concentrations in chickens ranged from 0.1 (muscle) to 70.2 µg/kg (liver). Carry-over ratios from feed to edible tissues were between 0.1 and 2% in pigs, and between 0.1 and 6.9% in chickens, suggesting a low contribution of pig and poultry tissue-derived products towards the total dietary CIT intake for humans.

## 1. Introduction

Mycotoxins are structurally diverse toxic fungal secondary metabolites. Citrinin (CIT) is a polyketide mycotoxin, mainly produced by *Aspergillus*, *Monascus* and *Penicillium* species [1]. CIT has demonstrated nephrotoxic effects and is frequently detected in a variety of foodstuffs, such as cereals and cereal-based products, nuts, herb and spices, causing a potential threat for human and animal health [2,3,4,5,6]. In feed, CIT incidence is also frequent. In a recent study, analysis of 90 chicken and pig feed samples in Belgium revealed a prevalence of almost 50%. The highest detected CIT concentration was 3.9 µg/kg feed [7]. In Russia, 1743 feed samples were analyzed for CIT, demonstrating a highest prevalence of 30% in sunflower oilseed meals. The highest detected concentration of CIT was 998 µg/kg in barley [8]. Several in vitro and in vivo studies have showed the toxic effects of CIT [9]. In general, CIT-mediated toxicity is considered to be related to oxidative stress and modified anti-oxidative enzymatic defenses [10,11,12,13,14,15,16]. Nephrotoxicity is a well-known toxic effect of CIT, as demonstrated in several species, such as dogs, rabbits, chickens, rats and pigs [11,17,18,19]. The median lethal dose (LD_50_) ranges from 19 mg/kg to 134 mg/kg body weight (bw), assessed after intravenous and oral administration in rabbits, respectively [20,21].

CIT toxicity is of particular interest in pigs and poultry, as they are economically important livestock. Moreover, their diet consists mainly of cereals and other grains, which are major sources of CIT. For pigs, few studies on CIT toxicity exist. Sandor et al. [22] did not observe any clinical signs of toxicity at a dose of 0.02 mg/kg bw per day. This was in accordance with the 90-day toxicity study of Lee et al., in which no nephrotoxicity was observed after administration of 200 µg/kg CIT per day through *Monascus* fermented products (equivalent to 0.02 mg/kg bw per day) [23]. Hence, a no observed adverse effect level (NOAEL) of 0.02 mg/kg bw per day is currently advised for pigs and 0.2 µg/kg bw per day for humans, taking a safety factor of 100 into account [24]. For poultry, no NOAEL is currently available. Very few LD_50_ values were reported in the literature, except for turkey poults and ducklings (56 and 57 mg/kg bw per day respectively) [25]. Feed containing 65 mg/kg of CIT did not cause any toxic effects in broiler chickens [26]. However, in another study, moderate effects have been observed in broiler chickens administered 33 mg CIT/kg feed (equivalent to 1.7 mg/kg bw per day) [27]. In this 6 weeks exposure study, broilers were fed with 0, 33, 65, 130 and 260 mg CIT/kg feed, revealing mottled livers, enlarged kidneys and hemorrhagic jejunums in the two highest dose groups. No mortality nor macroscopic lesions were observed at lower dose levels. At all dietary levels, lymphocyte and eosinophil infiltrations of the liver, kidneys and pancreas were observed.

Meerpoel et al. [28] demonstrated a moderate absolute oral bioavailability of around 40% in pigs, while absorption was complete in broiler chickens. Moreover, CIT was more rapidly absorbed in pigs (Average T_max_ of males and females = 3 h) compared to broilers (average T_max_ of males and females = 7 h). Furthermore, elimination half-lives differed significantly between species (T_1/2el_ of 18 h in pigs versus 5 h in broiler chickens after oral administration). Differences in toxicokinetics could be reflected in species-dependent sensitivity to toxicity. A lower oral bioavailability could imply a higher concentration in the intestines, resulting in impaired intestinal health. Furthermore, long persistence in the body and the relatively high distribution volume observed in broilers (3 L/kg) could possibly lead to carry-over in edible tissues such as kidneys, liver and eggs. In laying hens, carry-over to eggs and muscles was indeed demonstrated by Abdelhamid and Dorra [29], reporting CIT concentrations ranging from 6.2 to 10.6 µg/kg after a 6 week feeding trial with 0.1 mg CIT/kg feed. After a withdrawal period of 2 weeks, no CIT could be detected in any of the tissues. 

It is clear that carry-over of CIT is possible, and moderate histopathological effects could be observed at low concentrations, but the number of studies regarding carry-over of CIT is limited. As CIT shows different toxicokinetic characteristics in pigs and chickens, it is important also to perform toxicity studies, since different outcomes could be expected. Since CIT has a longer residence time in pigs compared to broiler chickens, the longer exposure time could lead to higher toxic effects in pigs. On the other hand, the distribution volume of CIT was tenfold higher in broiler chickens, indicating that higher concentrations of CIT could be obtained in organs/tissues. Although acute and short-term toxicity studies of several species have been performed in the past, the European Food Safety Authority (EFSA) stated in its scientific opinion on CIT that there is still missing data, especially concerning the transfer of CIT into organs [24]. Moreover, high doses were used in feeding trials (up to 500 mg CIT/kg feed in pigs and chickens, respectively [30,31]), but it is not clear whether lower CIT doses also cause (nephro)toxic effects. Currently, no toxicity and carry-over studies in pigs and broiler chickens exist, while these species are important livestock animals and accurate risk assessment of CIT for these species is therefore necessary. Moreover, information regarding the biotransformation into the less toxic dihydrocitrinone (HO-CIT) metabolite in animals is limited, while a previous toxicokinetic study in pigs and chickens demonstrated its presence in high concentrations in the blood [28]. It is not clear whether this metabolite is also transferred into the edible tissues. The aim of this study is therefore to develop and validate a quantitative ultra-high performance liquid chromatography-tandem mass spectrometry (UHPLC-MS/MS) method for determination of residues of CIT and its metabolite HO-CIT in kidney, liver, muscle (breast muscle of chickens and back muscle of pigs), skin with adhering fat, and eggs, in order to calculate possible carry-over of CIT from feed to edible tissues of pigs, broilers and layers after chronic dietary intake of CIT during 3 weeks, and to study its tissue depletion after cessation of dietary CIT intake. During the 3-weeks CIT intake, plasma concentrations of CIT were also evaluated. Furthermore, possible histopathological effects of CIT contaminated diets for 3 weeks on the kidneys, livers and intestines of pigs, broiler chickens and laying hens were studied.

## 2. Results

### 2.1. Method Validation for Edible Tissue

The UHPLC-MS/MS method for the quantification of CIT in kidney, liver, muscle, skin with adhering fat, and eggs was successfully validated. Results are summarized in the Supplementary Materials (Tables S1–S3). Limits of quantification (LOQ’s) ranged between 0.1 and 5 and 0.5 and 10 µg/kg for CIT and HO-CIT, respectively. A good linearity was observed, with correlation coefficients >0.99 and satisfactory goodness-of-fit coefficients of <20% (Table S1). Injection carry-over during analysis was not observed, since no peak higher than the limit of detection (LOD) was detected in the retention time range of CIT, HO-CIT or ^13^C_13_-CIT after injection of neat solvent. The results for apparent recovery and precision fell within the acceptance criteria, with only few small exceptions (Tables S2 and S3). 

### 2.2. Carry-over and Depletion Studies

No clinical signs in any of the animals in the control and experimental group were observed during the 3 weeks of CIT administration Daily feed intake was complete as no leftover feed was observed in the stables. Table 1 summarizes the CIT concentrations in the edible tissues at day 22, 24 h after the last feeding moment with CIT contaminated feed. In the control samples, no CIT nor HO-CIT was detected. Tissues that were collected from pigs, broilers and layers on the last day of CIT administration (i.e., just before the feed was switched to control feed) contained CIT in a concentration above the LOQ. This indicates carry-over of CIT to edible tissues, to which humans can possibly be exposed after consumption, although to a limited extent. The carry-over ratio in pig tissues ranged from 0 (no CIT detected) to 2% (highest observed concentration in liver), and from 0 to 6.9% (broiler liver) in chickens (Table 2). 

Rapid depletion occurred in pig tissues. At day 25, no CIT was detected. In contrast, concentrations higher than the LOQ were still observed at 3 days after CIT withdrawal in layers and broilers, especially in the highest dose groups. Depletion profiles in tissues for all species are given in the Supplementary materials (Figures S1–S11). 

**Figure 1:**
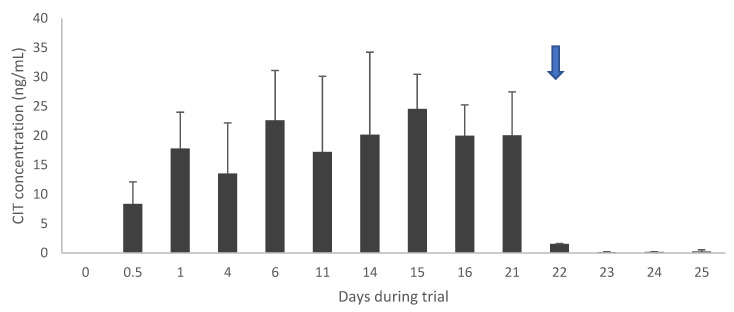
CIT concentrations in plasma at the start of the feeding trial (day 0) until day 21 in pigs (n = 16). Day 22–25 (arrow) represents concentrations after administration of control feed, hence representing the depletion of CIT (n = 2 per day).

**Figure 2:**
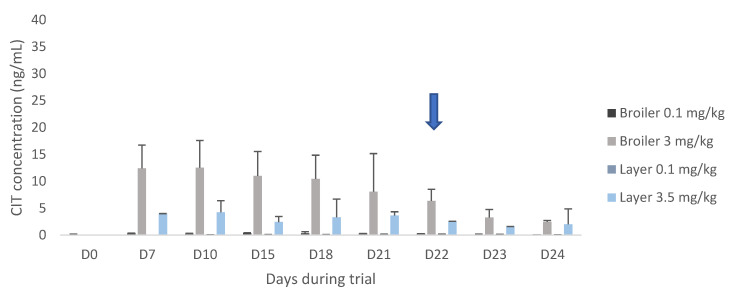
Citrinin (CIT) plasma concentrations at the start of the feeding trial (day 0) until day 21 in broiler chickens and laying hens (n = 8 per dose and species). Eight layers received CIT contaminated feed at 0.1 mg/kg, while the other 8 animals received CIT contaminated feed in a concentration of 3.5 mg/kg. Day 22–24 (arrow) represents concentrations after administration of control feed, hence representing the depletion of CIT (n = 2 per day, species and dose).

After 3 weeks, CIT administration was ceased, and CIT plasma concentrations rapidly declined from day 22 onwards in both pigs and broilers (Figure 1 and Figure 2). However, concentrations in layer hens in the high dose group did not decline but remained rather constant (Figure 2). HO-CIT concentrations were measured in plasma and edible tissue (Table 3). The highest concentration of the metabolite was found in the liver of pigs (80.12 µg/kg) and in the kidneys of broilers (118.19 µg/kg).

### 2.3. Histopathological Studies

**Figure 3:**
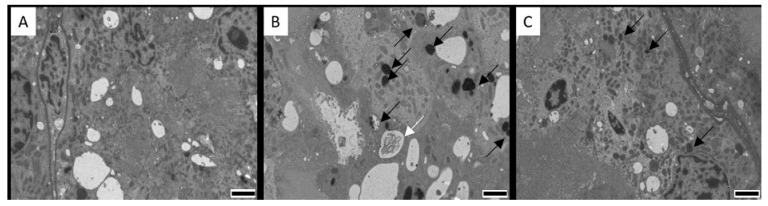
Cortical renal cells from the proximal convoluted tubules from (**A**) control pig; (**B**) pig in the experimental group exposed to 1 mg citrinin (CIT)/kg feed during 21 days; (**C**) pig in the experimental group 3 days after cessation of exposure to 1 mg CIT/kg feed during 21 days. Degenerated mitochondria are present in (**B**) (black arrows). Note a large vacuole with digested material (white arrow). Degenerated mitochondria are still present in (**C**) to a lesser extent. Magnification 2500.×. Scale bars: 2 µm.

**Figure 4:**
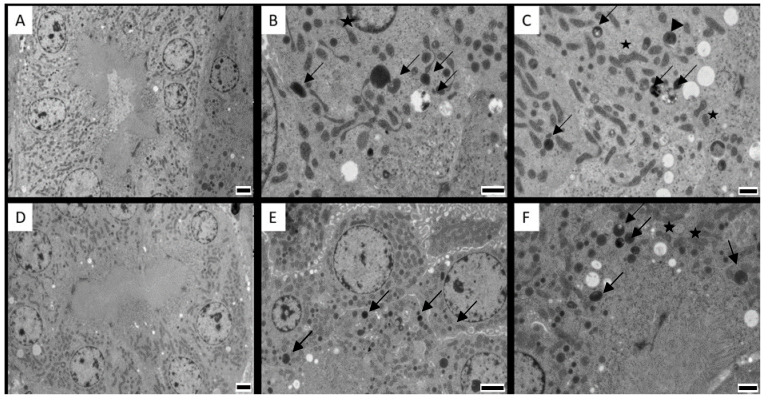
Cortical renal cells from proximal convoluted tubules from (**A**) control broiler chicken; (**B**) broiler chicken in the experimental group exposed to 0.1 mg citrinin (CIT)/kg feed during 21 days; (**C**) broiler chicken in the experimental group exposed to 3 mg CIT/kg feed during 21 days; (**D**) control laying hen; (**E**) laying hen in the experimental group exposed to 0.1 mg CIT/kg feed during 21 days; (**F**) laying hen in the experimental group exposed to 3.5 mg CIT/kg feed during 21 days. Degenerated mitochondria are present in all treated animals (arrows); note remaining cristae inside the vesicles (arrowhead). The stars indicate examples of degenerating mitochondria, with increased space in between the cristae. Magnification: 2500×. Scale bars: (**A**,**D**,**E**):2 µm; (**B**,**C**,**F**): 1 µm.

**Figure 5:**
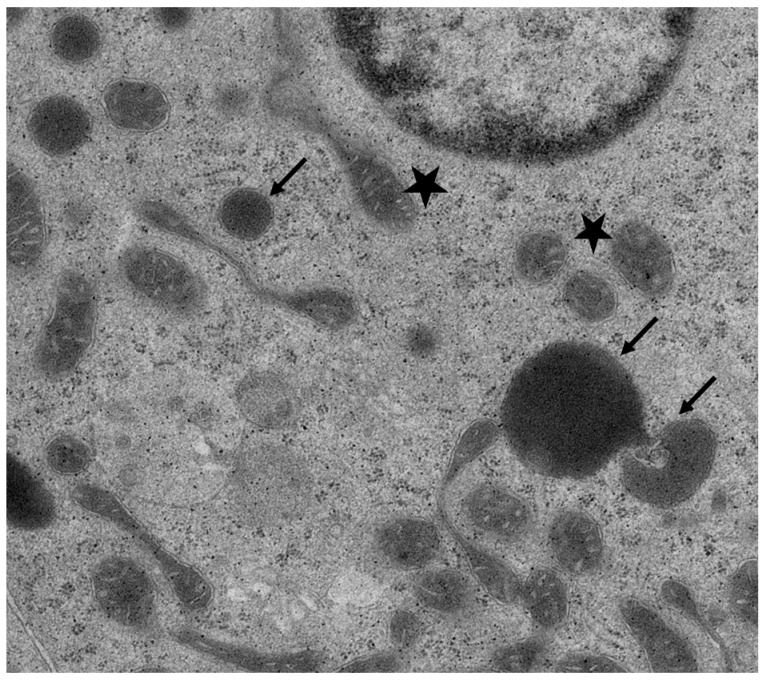
Magnified photograph of Figure 4B, showing clear spaces between cristae of mitochondria (stars) and mitochondrial degeneration (arrows).

Histopathological examination with light microscopy of the kidneys, liver, duodenum and jejunum did not reveal abnormalities nor major differences between the CIT fed groups and the control group (Supplementary Figures S12–S20). Images from jejunum of layers and duodenum of both species was not available due to technical problems. Since CIT is highly nephrotoxic, aliquots from the cortical region of kidney tissue were prepared for TEM examination. No ultrastructural damage was observed. However, images from the cortex region of the kidneys (mainly proximal convoluted tubule cells) showed mitochondria that were changed in shape and were slightly swollen, marked by an increased space between cristae in pigs and chickens of the highest dose group (Figure 3 and Figure 4, with a detailed TEM image in Figure 5).

**Figure 6:**
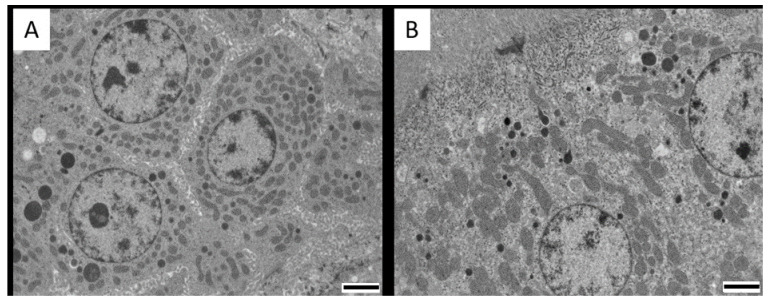
Cortical renal cells from proximal convoluted tubules from (**A**) broiler chicken in the experimental group 3 days after cessation of exposure to 3 mg citrinin (CIT)/kg feed during 21 days; (**B**) laying hen in the experimental group 3 days after cessation of exposure to 3.5 mg CIT/kg feed during 21 days. Note degenerated mitochondria in (**A**), implying recovery from oxidative stress, and the high number of degenerated mitochondria in (**B**). Magnification: 2500×. Scale bars: 2 µm.

The number of degenerated mitochondria differed significantly between the control pigs and pigs receiving CIT contaminated feed (*p* = 0.01), while there was no significant difference between the controls and pigs after cessation of CIT exposure (*p* = 0.33). Likewise, a significant difference was observed in broiler chickens and laying hens from the highest dose groups, compared to controls (*p* = 0.001 for both). However, while there was also a significant difference between broilers from the low dose group and controls (*p* = 0.003), no statistically significant difference was demonstrated between laying hens from the low dose group and controls (*p* = 0.8). After 3 days of cessation of CIT administration, the broiler chickens and laying hens from the highest dose group still had a significantly higher number of degenerated mitochondria compared to the control (*p* = 0.001 for both) (Figure 6).

## 3. Discussion

Carry-over of CIT to edible tissues was demonstrated in livestock, indicating that humans can possibly be exposed to CIT after meat consumption, although to a limited extent. In a recent study, chicken breast meat sold on the Belgian market was analysed for CIT, and a maximum concentration of 0.2 µg/kg was found (unpublished data). Furthermore, no CIT was detected in pork derived products. Therefore, carry-over CIT into fresh edible chicken tissue was a plausible reason for CIT contamination, but the risk is rather low. The carry-over ratio in pig tissues ranged from 0 (no CIT detected) to 2% (highest observed concentration in liver), and from 0 to 6.9% (broiler liver) in chickens. Relatively high CIT concentrations were found in liver tissue of chickens. Remarkably, in both broilers and layers, CIT carry-over ratios were higher in the low dose group (0.1 mg/kg feed), namely 0.1–6.9% and 0.2–4.3%, compared to the higher dose groups (3 and 3.5 mg/kg), namely 0.1–2.3% and 0.1–0.8%, respectively. This was especially the case for liver tissue and could possibly indicate a saturation in distribution. Similar observations were made in previous mycotoxin residue studies, in which higher carry-over factors were calculated for low contaminated feed, compared to feed with a higher mycotoxin content. Fraeyman et al. [32] reported higher carry-over factors for enniatin B in low-contaminated feed (135 µg/kg) versus high-contaminated feed (2.352 µg/kg) in broiler chickens. In another study, mean carry-over factors in pig liver for a 25% deoxynivalenol-contaminated wheat in the diet were higher than the 50% contaminated wheat. Lower accuracy at low mycotoxin concentrations may have contributed to this observation, although this was not the case in our study. The carry-over factors in the current study decreased from liver > kidney > skin and fat > muscle in pigs, from liver > skin and fat > kidney > muscle in broiler chickens and from liver > egg yolk > skin and fat > muscle > kidney in layer chickens. Considering the log P value of CIT (1.8), this rather hydrophobic compound tends to accumulate more in fatty tissues. Interestingly, mean concentrations differed between broilers and layers. Except for muscle tissue, CIT concentrations in tissues were higher in broilers compared to layers. Oral bioavailability is complete in broilers [28]; however, absorption data for CIT in layer hens is not available to our knowledge. It is possible that CIT is not fully absorbed in layer hens, as is the case also in pigs (around 40%). A higher accumulation of CIT in broilers compared to layers was also observed for ochratoxin A (OTA), which is structurally related to CIT [33]. In pigs, carry-over experiments were done with OTA-contaminated feed [34]. From this study, carry-over ratios of 1% and 10% in liver and kidney, respectively, could be calculated. Furthermore, the occurrence of OTA in pig derived meat products after consumption of an OTA contaminated diet was studied by Dall’Asta et al. [35]. A carry-over ratio of 2% into pig muscle (ham) was derived from this study. Compared to our study, the carry-over ratios calculated for CIT are lower than those for OTA. Bozzo et al. [36] did not find OTA residues in eggs after administration to laying hens of food containing 100 µg/kg OTA. Administering the same feed concentration to broiler chickens revealed no CIT residues in breast muscle, but concentrations of 3.6 and 1.9 µg/kg were detected in kidney and liver tissue, respectively [37]. Consequently, carry-over ratios of 3.6% and 1.9% were calculated for kidneys and liver, respectively. In contrast, our study demonstrated a higher carry-over rate of CIT into broiler liver (6.9%) compared to the kidneys (0.2%), after administration of 0.1 mg CIT/kg feed. Considering the relatively low concentrations of CIT residues, a small contribution of pig and poultry tissue-derived products to the total dietary CIT intake for humans is suggested. CIT contaminated feed at 1 mg/kg resulted in concentrations of 0.62 to 20.26 µg/kg in edible tissues of pigs. An adult ingesting 100 g of pig liver, which is included in the standard food basket according to Commission Regulation (EU) 2018/782 [38], would be exposed to 0.03 µg/kg bw per day assuming a bw of 70 kg, which is still far below the NOAEL of 0.2 µg/kg bw per day. Moreover, mean CIT concentrations are usually lower than 1 mg/kg, since 91% of contamination data are left censored [24]. When feeding 3 mg CIT/kg feed to broilers, consumption of 100 g of chicken livers would lead to an exposure of 0.1 µg/kg bw per day, which is close to the NOAEL, especially when more is consumed. However, it is unlikely that chicken feed contains CIT concentrations as high as 3 mg/kg, as the maximum concentration found in Europe so far is below 1 mg/kg [7,24]. However, outside Europe, a study in India revealed a maximum CIT concentration in poultry feed of 4.8 mg/kg, which is even higher than the experimental concentrations in this study. Thus, monitoring of CIT in feed is recommended as CIT can reach edible tissues and hence end up in the food chain.

Based on literature data, high concentrations were expected in chicken muscle tissue, since Abdelhamid and Dorra [29] detected 10 µg/kg in breast muscle tissue of layers after administration of 0.1 mg/kg feed, corresponding to a carry-over ratio of 10%. In contrast, this study revealed lower concentrations and a low carry-over ratio of 0.1% for breast muscle. Also, regarding eggs, the aforementioned study reported a carry-over ratio of 10% and 6% for egg yolk and egg white, respectively. In contrast, CIT was not detected in egg white in this study, whereas low concentrations were found in egg yolk in both dose groups, yielding carry-over ratios of 0.6% and 0.03%. However, it should be noted that there were differences in duration of the trial (6 weeks versus 3 weeks in the present study) and the age and breed of layers used (13 weeks-old Egyptian Mamourah breed versus 20 weeks-old Isa White breed in this study). Moreover, the sample size per chicken group was small as only two animals per group were euthanized at day 21, i.e., when steady-state concentrations of CIT were reached and before the test feed was replaced by control feed. Rapid depletion occurred in pig tissues. In contrast, concentrations higher than the LOQ were still observed at 3 days after CIT withdrawal in layers and broilers, especially in the highest dose groups. In plasma, steady-state concentrations were reached within 4 days in pigs, and within 1.5 days in broiler chickens. This reflects the differences in plasma elimination half-lives for CIT in pigs and broiler chickens after oral administration (average T_1/2el_ for males and females of 18 versus 5 h, respectively). After 3 weeks, CIT administration was ceased, and CIT plasma concentrations rapidly declined from day 22 onwards in both pigs and broilers. However, concentrations in layer hens in the high dose group did not decline but remained rather constant. Using the depletion data, a T_1/2el_ of approximately 6 h was roughly calculated for pigs, while the T_1/2el_ for broilers and laying hens was estimated to be 24 h and 36 h, respectively. Interestingly, earlier observations from a toxicokinetic study in broilers, where CIT was administered in a single oral dose of 0.25 mg/kg bw, revealed a plasma elimination half-life of 5 h and no CIT was detected after 72 h post administration [28]. The reason why CIT was still detected after 4 days, could be a build-up of the toxin in the tissues after 3 weeks of contaminated diet, while the toxicokinetic study with a single dose of CIT would probably not result in accumulation in the tissues. Likewise, the calculated T_1/2el_ for pigs is not in accordance with the previously performed toxicokinetic study with an administered P.O. dose of 0.05 mg/kg bw (T_1/2el_ = 18 h). HO-CIT concentrations were also measured in plasma and edible tissue. The metabolite was found in higher concentrations than its parent compound CIT in all species studied. This indicates that CIT was extensively metabolized into HO-CIT. The same trends concerning CIT were observed for HO-CIT comparing broilers and layer hens. A human toxicokinetic study also reported higher HO-CIT concentrations compared to CIT in urine, while no HO-CIT could be detected in plasma [39]. In accordance with CIT, HO-CIT concentrations declined rapidly in pigs, while considerable amounts of HO-CIT were still present in poultry tissues during the withdrawal period. 

No ultrastructural damage was observed in kidneys, liver and intestines. However, changes in renal mitochondria were visually observed. Although these observations are difficult to interpret without further confirmation, it is of interest to point out that mitochondrial swelling and misshapen appearance are consistent with the concept that the lesions of this organelle are crucial in the mechanism of CIT toxicity. Studies have reported multiple effects of CIT on mitochondrial function, impairing mitochondrial respiration [40,41]. At higher doses, mitochondrial alterations are clearer, including peripheral condensation and pleomorphisms [40,42,43]. The swelling and misshapen appearance of the mitochondria might be caused by the accumulation of intracellular water as a result of toxic stress [44]. Mitochondrial condensation may lead to respiratory stimulation or altered membrane permeability and altered mitochondrial configuration. Mitochondria were also involved in CIT-induced apoptosis in HL-60 cells, as cytochrome-c (a component of the mitochondrial electron transfer chain) is released from the mitochondria, which plays a key role in mitochondria dependent apoptosis. [14]. These mechanisms were also described for OTA, causing apoptosis related to the loss of mitochondrial membrane potential [45]. Furthermore, similar histopathological changes in mitochondria related to OTA exposure in quail and laying hens were reported [40,46]. In all treated animals and dose groups, degeneration of mitochondria was observed in the renal cortical cells compared to controls (Figure 3 and Figure 4). It is plausible that cells prepared for apoptosis due to oxidative stress caused by CIT. In pigs, no significant difference was observed between the number of degenerated mitochondria in the controls and pigs after cessation of CIT exposure (*p* = 0.33). This indicates a possible recovery of renal cells when CIT exposure was ceased. However, after 3 days of cessation of CIT administration, the broiler chickens and laying hens from the highest dose group still had a significantly higher number of degenerated mitochondria compared to the control. Therefore, it seems that recovery from mitochondrial damage is a slower process in chickens compared to pigs. Interestingly, depletion of CIT was slower in broiler chickens and laying hens compared to pigs, which could explain the presence of degenerated mitochondria even after cessation of CIT administration

No severe toxic effects were expected, since a previous study of broiler chickens fed a diet containing 65 mg/kg feed did not reveal any clinical signs of toxicity. In pigs, no clinical sigs of toxicity were observed at 20 µg CIT/kg bw per day (22), but our study demonstrates slight changes in mitochondria after administration of 50 µg CIT/kg bw per day in pigs, and 10/250 µg/kg bw in broiler chickens and laying hens.

## 4. Conclusions

This study demonstrates that the presence of CIT in animal feed can lead to its transfer into edible tissues, although with low carry-over rates. Although it is unlikely that high amounts of CIT will be found in animal feed, it is shown that this toxin tends to accumulate in tissues of animal origin, and a contribution to the total dietary CIT intake of humans is plausible. Depletion of CIT in plasma and tissues was rapid in pigs, but slow in chickens, especially in layers. Furthermore, it was demonstrated that exposure to 1 mg CIT/kg feed for pigs, 0.1 and 3 mg/kg for broiler chickens and 0.1-3.5 mg/kg for layer hens during 3 weeks does not cause toxic effects in kidneys, liver, jejunum and duodenum, after histopathological inspection using light microscopy. However, the present study demonstrated mitochondrial changes, using transmission electron microscopy, which might play a vital role in the mechanism of nephrotoxicity. Changes were already visible at relatively low doses of CIT.

## 5. Materials and Methods

### 5.1. Chemicals and Reagents

CIT was purchased from Fermentek (Jerusalem, Israel) and HO-CIT (Figure 1) was obtained from Analyticon (Potsdam, Germany). Stock solutions of 1 mg/mL were prepared in methanol (MeOH). A dissolved standard of ^13^C-labelled CIT (^13^C_13_-CIT) as internal standard (IS) in acetonitrile (ACN) was purchased from Biopure-Romer Labs (Tulln, Austria), with a concentration of 10.6 µg/mL. All stock solutions were stored at −20 °C. From the individual stock solutions, a CIT/HO-CIT standard mixture of 10 µg/mL each and a ^13^C_13_-CIT solution of 100 ng/mL were prepared in MeOH. A Milli-Q SP Reagent water system (Merck Millipore, Darmstadt, Germany) was used to obtain ultrapure water. MeOH (LC-MS grade, 99.95%), ethyl acetate (EtAc, 99%), hydrochloric acid (HCl, 37%) were purchased from BioSolve BV (Valkenswaard, The Netherlands), ACN (HiPerSolv Chromanorm HPLC grade, 99.9%) was acquired from VWR International (Leuven, Belgium), whereas sodium chloride (NaCl, 99.9%), ammonium acetate (98%) and glacial acetic acid (HAc, 100%) were purchased from Merck. Magnesium sulfate (MgSO_4_, 99.5%) was procured from Sigma-Aldrich (Bornem, Belgium).

### 5.2. Quantification of CIT and HO-CIT in Plasma and Edible Tissues

#### 5.2.1. Plasma

A previously developed and validated method from Meerpoel et al. [28] was used for analysis of CIT and HO-CIT in pig and chicken plasma. In brief, sample preparation consisted of deproteinization of plasma (250 µL) with ACN (750 µL), followed by centrifugation (8517× *g*, 10 min, 4 °C). The ACN phase was evaporated under a gentle nitrogen stream at 40 °C and the dry residue was reconstituted in 250 µL of MeOH/water (50/50, *v/v*). The limit of detection (LOD) and limit of quantification (LOQ) were 0.05 and 0.1 ng/mL for CIT and 0.01 and 0.1 ng/mL for HO-CIT, respectively.

#### 5.2.2. Breast and Back Muscle, Kidney, Liver, Skin with Adhering Fat

Samples of breast, back muscle, kidney, liver and skin were minced, homogenized using a meat grinder from Moulinex and stored at −20 °C until extraction. In a 50 mL extraction tube, 1.00 ± 0.05 g of homogenized sample was weighed. The samples were spiked with CIT/HO-CIT to construct a matrix-matched calibration curve at the following concentrations: 0.1–0.5–1–5–10–50–100–200 µg/kg. To each sample, ^13^C_13_-CIT was added at 5 µg/kg. After an equilibration period of 15 min, 3 mL of a HCl 1M solution was added and samples were left at room temperature for another 15 min. Next, 5 mL of EtAc was added, followed by shaking for 30 min using an overhead shaker (Trayster Digital, Ika-Werke, Staufen, Germany). Subsequently, 1.5 ± 0.05 g of MgSO_4_ and 0.4 ± 0.04 g of NaCl were added. Samples were first agitated by hand for 30 s to avoid aggregation of the salts, followed by shaking using the overhead shaker for 3 min. Next, samples were centrifuged for 5 min at 3676× *g*. An aliquot of 1 mL supernatant was transferred and evaporated to dryness under a gentle stream of nitrogen at 40 °C. Extracts were reconstituted in 0.2 mL of injection solvent (MeOH/water, 50/50 *v/v*), vigorously vortexed for 1 min and subjected to ultracentrifugation for 10 min at 9167× *g*. Finally, extracts were transferred into autosampler vials for analysis. 

#### 5.2.3. Egg Yolk and Egg White

A 4.00 ± 0.02 g homogenized sample was weighed in a 50 mL extraction tube. The samples were spiked to construct a matrix-matched calibration curve at the following CIT/HO-CIT concentrations: 0.1–0.5–1–5–10–50–100–200 µg/kg. To each sample, ^13^C_13_-CIT was added at a concentration of 5 µg/kg After leaving the samples 30 min for equilibration, 10 mL of an acidified saline solution was added, consisting of 10% (m/v) NaCl in acidified water (1.6% HCl in H_2_O/HAc (99/1, *v/v*)), followed by 20 mL of extraction mixture consisting of EtAc, ACN and HAc (75/24/1, *v/v/v*). Samples were extracted for 1 h at room temperature using an overhead shaker (Agilitec, J. Toulemonde and Cie, Paris, France). Subsequently, 6.0 ± 0.2 g of MgSO_4_ and 1.5 ± 0.1 g of NaCl were added. Samples were first agitated by hand for 30 s to avoid aggregation of the salts, followed by shaking using the overhead shaker for 3 min. Next, samples were centrifuged for 5 min at 3676× *g*. An aliquot of 1 mL supernatant was transferred and evaporated to dryness under a gentle stream of nitrogen at 40 °C. Extracts were reconstituted in 0.2 mL of injection solvent (MeOH, water 50/50 *v/v*), vigorously vortexed for 1 min and subjected to ultracentrifugation (Ultrafree^®^-MC centrifugal device; Millipore, Bedford, MA, USA) for 10 min at 9167× *g*. Finally, extracts were transferred into autosampler vials for analysis.

#### 5.2.4. Ultra-high Performance Liquid Chromatography Tandem Mass Spectrometry

The obtained extracts were analysed using a previously developed UHPLC-method [7].

### 5.3. Method Validation for Edible Tissues

The developed method was in-house validated, using spiked blank liver, kidney, skin with adhering fat, muscle, egg yolk and egg white samples obtained from healthy, untreated broiler chickens or pigs. A 3-day validation was performed on pig muscle tissue, while method performance was evaluated on skin with adhering fat, liver, kidney and eggs by a 1-day validation. Linearity, carry-over, apparent recovery, within-run and between-run precision, LOQ and LOD were determined in accordance with the recommendations and guidelines defined by the European Commission [47].

Linearity of a matrix-matched calibration curve was evaluated using the coefficient of determination (R²) and confirmed by calculation of goodness-of-fit (g) (%), which takes into account the difference between nominal value of the calibration curve and the calculated concentration (Equation (1)).

(1) $$g=\;\sqrt{\frac{\sum {\left(\%\;\mathrm{deviation}\right)}^{2}}{n-1}}\;\mathrm{With}\;\%\;\mathrm{deviation}\;=\;\frac{\mathrm{calculated}\;\mathrm{concentration}-\mathrm{nominal}\;\mathrm{value}\;}{\mathrm{nominal}\;\mathrm{value}}\;\times \;100$$

Apparent recovery (%) was determined in triplicate as the ratio of the measured concentration divided by the actual (spiked) concentration, evaluated at 3 levels (0.1, 10 and 200 µg/kg). The acceptance criteria for apparent recovery were: 50% to 120%, 70% to 110% and 80% to 110% for concentration levels ≤1 µg/kg, between 1 and 10 µg/kg and > 10 µg/kg, respectively. Within-run and between-run precision were determined by calculating the residual standard deviation (RSD) in % from 6 spiked samples at 3 concentration levels (low, medium and high level) on 1 day (within-day, RSD_r_) and on 3 different days (between-day, RSD_R_). RSD values should be as low as possible, <30% considered as acceptable. The LOQ was established as the lowest point of the calibration curve that could be analysed with good accuracy and precision within the recommended ranges, as confirmed by analyzing six samples spiked at the lowest concentration level. The LOD was defined as the lowest concentration that could be detected with a signal-to-noise (S/N) ratio of ≥3. Absence of carry-over was evaluated by injecting neat solvent directly after the highest calibrator. If a peak was present within the same retention time range of CIT and HO-CIT (± 2.5%), it had to be below the LOD. 

### 5.4. Experimental Diets

Incurred CIT contaminated rice and wheat batches were in-house produced through the following steps. First, a biobank of available strains of *Monascus* sp. (n = 5), *Aspergillus niger* (n = 4), *A. alutaceus* (n = 4) and *Penicillium citrinum* (n = 2) from the “Mycothèque de l’Université Catholique de Louvain, MUCL” was constituted. The potential ability of CIT production by these strains was screened at small scale in wheat grains or rice grains (3 replicates of 20 g of grains per fungus) after adjusting the humidity by adding Milli-Q water to obtain a humidity of 24–25%. These were inoculated and cultivated for 3 weeks on sterilized aliquots (wheat or rice grains). Incubation was stopped by autoclaving at 121 °C for 20 min and samples were thereafter dried at 50–60 °C for 48 h, ground and sieved (< 250 µm), homogenized and stored at −20 °C until analysis by LC-MS/MS [48]. *Penicillium citrinum* MUCL 29781 was selected for CIT production in contaminated feed. Biomass of contaminated wheat grains (kg amounts) was then produced via cultivation of the selected fungus in the same conditions as described above. Control material was identically prepared by replacing the inoculum substrate with sterilized water.

#### 5.4.1. Pig Feed

CIT reference material (wheat flour) was prepared, checked, and found to be sufficiently homogenized with the final concentration of 404.0 ± 19.7 µg/g. Note that this material was checked to be free of mycotoxins such as aflatoxins B1, B2, G1, G2, T2-toxin, HT2-toxin, fumonisin B1, B2, B3, deoxynivalenol, zearalenone and ochratoxin A. CIT content in control material was 0.0003 µg/g and free of the other tested mycotoxins.

Before the reference material was mixed with commercial pig feed, the feed was first analysed for CIT with a validated UHPLC-MS/MS method [7] to make sure it was CIT free. Therefore, 4 subsamples of 500 g were randomly collected from the 200 kg batch, pooled and homogenized. No CIT was detected in the feed. To reach a concentration of 1 mg/kg feed, 500 g of the prepared reference material was mixed with 200 kg of pig feed (Biggistart Optimeel, AVEVE, Belgium) using a mechanical mixer. Feed concentrations correspond to a daily intake of 0.05 mg CIT per kg bw, assuming a daily feed intake of 1 kg and an average bw of 20 kg. The rationale behind this concentration is that the daily intake is identical to the dose used in our previously reported toxicokinetic study in pigs and broiler chickens [28]. Furthermore, the highest reported CIT concentration in grains intended for swine was 998 µg/kg [8]. Due to practical reasons, a lower dose group was not included, in contrast to the experimental groups of chickens.

#### 5.4.2. Chicken Feed

##### Broilers

Two wheat reference materials were prepared and checked to contain 2.0 ± 0.4 µg/g (batch 1) and 53.2 ± 3.3 µg/g (batch 2).

To reach a concentration of 0.1 mg/kg and 3 mg/kg feed, 2 kg of batch 1 and 2.25 kg of batch 2 was mixed respectively with 40 kg of broiler feed (Farm 2 Mash, Versele-Laga, Deinze, Belgium). The commercial feed was first checked for CIT content, revealing a low, unquantifiable concentration (>LOD of 0.3 µg/kg but < LOQ of 1 µg/kg. Feed concentrations correspond to a daily CIT intake of 0.01 and 0.25 mg per kg bw, assuming a daily feed intake of 200 g and an average bw of 2.25 kg. The rationale behind the concentration of 3/3.5 mg/kg feed is that the daily intake is identical to the dose used in our previously reported toxicokinetic study in pigs and broilers chickens [28]. Furthermore, a lower dose was used to assess histopathological effects and carry-over at lower CIT concentrations equal to the maximum allowed limit of 100 µg/kg for ochratoxin A in poultry feed, which is structurally related to CIT and therefore might exert similar effects [49]. For CIT, there is currently no legal limit for feed.

##### Layers

Additionally, two batches of wheat reference materials were produced to contain 2.0 ± 0.4 µg/g (batch 1) and 71.6 ± 2.0 µg/g (batch 2). 

To reach a concentration of 0.1 mg/kg and 3.5 mg/kg feed, 1 kg of batch 1 and 1 kg of batch 2 was mixed respectively with 20 kg of broiler feed (Gold 4 Mash, Versele-Laga, Deinze, Belgium). The commercial feed was free of CIT. Feed concentrations correspond to a daily CIT intake of 0.01 and 0.25 mg per kg bw, assuming a daily feed intake of 177 g and an average bw of 1.66 kg. These intakes are the same as the intakes used for broiler chickens.

#### 5.4.3. Control Feed

The same commercial feed used to prepare the contaminated diets was used as control feed. The pig and layer feed did not contain CIT, while traces of naturally occurring CIT (<LOQ) were detected in the broiler feed. 

### 5.5. Feeding Trials

The animal experiments were approved by the Ethical Committee of the Faculty of Veterinary Medicine and the Faculty of Bioscience Engineering of Ghent University (EC 2017/105). All animal experiments were performed according to Directive 2010/63/EU [50] and the Belgian Royal Decree on the protection of laboratory animals of 29 May 2013 [51].

#### 5.5.1. Pigs

Twenty conventional pigs (hybrid sow x Piétrain boars, 10 males and 10 females, 6 weeks old, 12 ± 1.1 kg bw) were housed according to Directive 2010/63/EU [50] and the Belgian Royal Decree on the protection of laboratory animals of 29 May 2013 [51] at the Faculty of Veterinary Medicine of Ghent University in Merelbeke (Belgium). For 1 week, the animals were acclimatized and socialized in the stables. Water and control feed were given ad libitum. After 1 week, the pigs were randomly divided into 8 small groups of 2 animals per group (1 male, 1 female) and each group was housed in a separate pen of >2 m^2^. Four pigs were kept in a separate pen and served as control. For 3 weeks, the pigs received CIT contaminated feed (1 mg/kg) in an amount according to their body weight [52], once daily at a fixed time in the morning. Drinking water was available ad libitum. The feed troughs were monitored daily to control feed intake and to check whether the feed was completely consumed by the pigs. At several days at a fixed time in the afternoon (5 h after feeding), a blood sample of approximately 3 mL was drawn from the jugular vein by direct venipuncture using a BD vacutainer^®^ containing ethylenediaminetetraacetic acid (EDTA). The collected blood samples were centrifuged (3676× *g* for 10 min) within 2 h after collection and plasma was stored at −20 °C. After 3 weeks (day 21), 8 pigs (2 animals from 4 subgroups) were euthanized via intravenous administration of an overdose of pentobarbital (Release^®^, Ecuphar, Oostkamp, Belgium), while the remaining pigs received again control feed ad libitum. From the euthanized animals, blood, both kidneys, liver, muscle tissue (back muscle, approximately 250 g), duodenum, jejunum and skin with adhering fat were collected and immediately prepared for CIT residue analysis and histopathological investigation. For the residue analysis, the collected organs were stored at −20 °C within 1 h. Procedures for histopathology are described in Section 5.7. Each consecutive day and during 4 days after cessation of CIT exposure (day 22–25), the procedure was repeated on 2 pigs and 1 control pig. The total duration of the tissue collection was 5 days. 

#### 5.5.2. Broilers and Layers

Twenty broilers (Ross 308, 10 males and 10 females, 3 weeks old, 0.8 ± 0.5 kg bw) and 20 layers (Isa White, 20 weeks old, 1.9 ± 0.4 kg bw) were housed according to Directive 2010/63/EU [50] and the Belgian Royal Decree on the protection of laboratory animals of 29 May 2013 [51] at the Faculty of Veterinary Medicine of Ghent University in Merelbeke (Belgium). For 1 week, the animals were kept all together but separate per species and acclimatized in the stables. Water and control feed were given ad libitum. After 1 week, the chickens were randomly divided into 4 small groups of 4 animals (2 males, 2 females for broilers) per group and each group was housed in a separate pen of > 2m².Four broilers and four layers were kept in a separate pen and served as control. For 3 weeks, animals were exposed through the feed to either a low (8 animals per species) or a high (8 animals per species) concentration of CIT. More specifically, 2 groups of 4 broilers received feed at 0.1 mg CIT/kg feed. The other 2 groups of 4 broilers received feed contaminated at 3 mg CIT/kg feed. Likewise, the laying hens received either 0.1 mg CIT/kg feed or 3.5 mg CIT/kg feed in an amount according to their body weight [53,54]. Feed intake was monitored daily, to ensure all feed was consumed. Eggs from the layers were collected daily, coded by day of collection and stored at 4 °C. At several days, a blood sample of approximately 1 mL was drawn from the leg vein by direct venipuncture using BD vacutainers^®^ containing EDTA. The collected blood samples were centrifuged (3676× *g* for 10 min) and the plasma was stored at −20 °C. After 3 weeks, 2 experimental animals and 1 control animal per group were euthanized (day 21), while the remaining animals again received control feed. From the euthanized animals, blood, kidneys, liver, breast muscle tissue (approximately 100 g), duodenum, jejunum and skin with adhering fat were collected and immediately prepared for CIT residue analyses and histopathological investigation. For the residue analysis, the collected organs were stored at −20 °C within 1 h. Procedures for histopathology are described in Section 5.7. Each consecutive day, and during 3 days after cessation of CIT exposure (day 22–24), the procedure was repeated on two chickens per group and 1 control animal.

### 5.6. Carry-Over and Depletion Studies

The collected tissues during the animal trials (eggs, kidney, liver, muscle tissue and skin with adhering fat) were extracted and analyzed using the validated UHPLC-MS/MS method described above. Carry-over rate (%) was defined as the ratio of the mean CIT concentration in the tissues determined at day 21 and the concentration in feed, multiplied by 100.

### 5.7. Histopathological Studies

#### 5.7.1. Light Microscopy

Samples of kidneys, liver, duodenum and jejunum were prepared. From the cortex region of each kidney, 4 samples of 0.5 cm³ were taken (n = 8 per animal). The livers were divided into 4 regions of equal size, and from each region, 2 samples of 0.5 cm³ were taken. From the intestines, aliquots of 2 cm from the jejunum (n = 2) and duodenum (n = 2) were rinsed with 0.9% normal saline. All samples were immediately fixed in phosphate buffered saline containing 4% formaldehyde and left for 24 h at room temperature. The samples were dehydrated in graded concentrations of ethanol. The fixed tissues were embedded in paraffin, after which tissue sections of 5 µm were stained with hematoxylin and eosin (H&E) using standard procedures [55]. Microscopic images were made using a BX61 Olympus microscope and cellSens software (Olympus Soft Imaging solutions GMBH, Munster, Germany). During evaluation, 10 areas equal in size from each microscopic specimen were randomly selected, in which signs of histological damages were counted and scored. Renal histological damage was quantified by the EGTI (Endothelial, Glomerular, Tubular, Interstitial) scoring system devised specifically for animal research on kidney tissue in the context of injury [56]. Intestinal histological damage was inspected based on Kolf-Clauw et al. [57]. The number of villi and the number of crypts, the morphology of enterocytes, the degrees of villi coalescence and autolytic changes of the tissue (oedema, necrotic debris, apoptotic cells) were considered. For liver damage evaluation, the histology activity index (HAI) based on Ishak et al. [58] was used. Parameters taken into consideration included acinar inflammation, focal or confluent necrosis, hemorrhages, the presence of lymphoid follicles, steatosis, hepatocellular dysplasia and karyomegaly.

#### 5.7.2. Transmission Electron Microscopy

For histopathological examination of kidney tissue, 3 aliquots (1 mm³) of the cortex region from both kidneys were transferred to individual test tubes containing Karnovsky fixative (2% paraformaldehyde and 2.5% glutaraldehyde in 0.1 M cacodylate buffer, pH 7.2) and stored at 4°C overnight. In the following sample preparation step, the samples were washed with 0.1 M cacodylate buffer and post-fixed with buffered 1% osmium tetroxide for 1.5 h at room temperature. Samples were rinsed afterwards with double-distilled water followed by dehydration in a graded series of ethanol (alcohol 10, 30, 50, 70 and 94% for 10 min each, absolute alcohol dehydrated with CuSO_4_ for 20 min, a 1:1 mixture of absolute alcohol and acetone for 10 min, and acetone for 30 min). Next, samples were impregnated in graded mixtures of acetone and epoxy resin (3:1, 1:1 and 1:3) for 2 h each, and in pure epoxy resin overnight. Afterwards, the embedded samples were left at 60 °C for 3 days to allow polymerization of the resin. Ultrathin sections (± 90 nm) were cut with a Leica Ultramicrotome (EM UC6) using a diamond knife (DIATOME, ultra 45°; 2.5 mm). Each sample was deposited on formvar coated grids. Samples were contrasted with 1% uranyl acetate and 1.33% lead citrate. Images were made by a Quemesa charge-coupled device camera (Olympus Soft Imaging solutions GMBH, Munster, Germany). Toxicity of CIT was assessed by a double blinded histological examination of the kidneys by an independent expert in histopathology. Size and shape of mitochondria were inspected. Furthermore, signs of apoptosis (presence of apoptotic bodies, pyknosis, karyorrhexis, blebbing of plasma membrane) were evaluated and the number of degenerated mitochondria were counted in 30 randomly selected cells per animal.

### 5.8. Statistical Analysis

For the histopathological samples, statistical differences between groups were determined with a two tailed, unpaired Student’s *t*-test. The significance level was set at *p* < 0.05. All statistical analyses were carried out using SPSS 26.0 (IBM, New York, NY, USA).

## Figures and Tables

**Table 1:** Mean steady-state citrinin (CIT) concentrations measured in plasma (ng/mL) and edible tissues (µg/kg) from pigs, broiler chickens and layers at day 22, 24 h after receiving CIT contaminated feed during 3 weeks at 0.1, 1, 3, 3.5 mg/kg feed, depending on species. Concentrations are given ± standard deviation. n = 8 for plasma of all groups, n = 2 for edible tissues of broilers and layers and n = 8 for edible tissues of pigs.

Animal Species	Pig	Broiler		Layer	
Feed CIT concentration	1 mg/kg	0.1 mg/kg	3 mg/kg	0.1 mg/kg	3.5 mg/kg
Plasma	20.78 ± 2.52	0.31 ± 0.05	11.26 ± 5.05	0.1 ± 0.05	3.08 ± 2.02
Muscle	0.62 ± 0.61	0.11 ± 0.35	1.81 ± 0.95	<LOQ	2.70 ± 0.70
Skin + fat	5.01 ± 3.14	0.74 ± 0.41	11.08 ± 5.01	0.20 ± 0.10	8.00 ± 3.37
Kidney	15.20 ± 9.56	0.23 ± 0.02	1.90 *	<LOQ	1.19 ± 0.105
Liver	20.26 ± 5.98	6.86 ± 1.37	70.21 *	4.29 ± 1.50	27.07 ± 7.18
Egg yolk	n.a.	<LOQ	<LOQ	0.58 ± 0.75	1.09 ± 0.72
Egg white	n.a.	<LOQ	<LOQ	<LOQ	<LOQ

* only 1 sample could be analysed; limits of quantification (LOQ’s) in pig: 0.1 ng/mL (plasma), 0.1 µg/kg (back muscle); 0.5 µg/kg (skin+fat, kidney, liver); LOQ’s in broiler and layer: 0.1 ng/mL (plasma), 0.1 µg/kg (breast muscle, egg white); 0.5 µg/kg (skin+fat, egg yolk); 1 µg/kg (kidney); 5 µg/kg (liver). n.a.: not applicable.

**Table 2:** Carry-over ratios (%) of CIT from feed to edible tissues from pigs, broiler chickens and layers 24 h after receiving CIT contaminated feed during 3 weeks at different concentration levels (0.1, 1, 3, 3.5 mg/kg feed, depending on species).

Animal Species	Pig	Broiler		Layer	
Feed CIT concentration	1 mg/kg	0.1 mg/kg	3 mg/kg	0.1 mg/kg	3.5 mg/kg
Muscle	0.1	0.1	0.1	n.d.	0.1
Skin + fat	0.5	0.7	0.4	0.2	0.2
Kidney	1.5	0.2	0.1	n.d.	0.03
Liver	2	6.9	2.3	4.3	0.8
Egg yolk	n.a.	n.d.	n.d.	0.6	0.03
Egg white	n.a.	n.d.	n.d.	n.d.	n.d.

n.d: not determined; n.a.: not applicable.

**Table 3:** Mean dihydrocitrinone (HO-CIT) concentrations (µg/kg) measured in plasma and edible tissues from pigs, broilers and layers 24 h after receiving CIT contaminated feed during 3 weeks at different levels (0.1, 1, 3, 3.5 mg/kg feed, depending on species). Concentrations are given ± standard deviation. n = 8 for plasma of all groups, n = 2 for edible tissues of broilers and layers and n = 8 for edible tissues of pigs.

Animal Species	Pig	Broiler		Layer	
Feed CIT concentration	1 mg/kg	0.1 mg/kg	3 mg/kg	0.1 mg/kg	3.5 mg/kg
Plasma	23.16 ± 8.18	5.02 ± 0.05	14.30 ± 2.69	2.27 ± 0.15	89.02 ± 17.69
Muscle	<LOQ	n.d.	9.70 ± 2.98	<LOQ	6.57 ± 1.97
Skin + fat	17.75 ± 14.43	2.67 ± 0.58	80.60 ± 15.64	<LOQ	38.01 ± 21.15
Kidney	53.23 ± 36.73	23.48 ± 12.76	118.19 *	<LOQ	18.64 ± 14.45
Liver	80.12 ± 12.95	n.d.	31.97 ± 5.49	<LOQ	8.27 ± 8.91
Egg yolk	n.a.	<LOQ	<LOQ	<LOQ	<LOQ
Egg white	n.a.	<LOQ	<LOQ	<LOQ	<LOQ

* only 1 sample could be analysed. LOQ’s in pig: 0.1 ng/mL (plasma), 1 µg/kg (back muscle); 0.5 µg/kg (liver); 5 µg/kg (kidney); 1 µg/kg (skin+fat). LOQ’s in broiler and layer: 0.1 ng/mL (plasma), 1 µg/kg (breast muscle, egg yolk); 5 µg/kg (kidney, skin+fat, egg yolk); 8 µg/kg (liver). n.a.: not applicable; n.d.: not detected.

## Supplementary Materials

The following are available online at https://www.mdpi.com/2072-6651/12/11/719/s1, Table S1. Validation parameters for citrinin (CIT) and dihydro-citrinone (HO-CIT) in edible tissue matrices for linearity (correlation coefficient R² and goodness-of-fit g), limit of detection (LOD), limit of quantification (LOQ), Table S2. Validation results for citrinin (CIT) and dihydro-citrinone (HO-CIT) for apparent recovery (R_app_), within-day (RSD_r_, n = 6) and between-day precision for muscle tissue (RSD_R_, n = 3 × 3) at 3 concentration levels for pigs. RSD_R_ was not calculated for other matrices, as a shortened one-day validation was applied, Table S3. Validation results for citrinin (CIT) and dihydro-citrinone (HO-CIT) for apparent recovery (R_app_), within-day (RSD_r_, n = 6) precision at 3 concentration levels for chickens. Figures S1–S10: Citrinin (CIT) or dihydro-citrinone (HO-CIT) concentrations measured in kidney, liver and skin with adhering fat tissue from pigs, broilers chickens and laying hens after euthanasia. At day 21, the CIT contaminated feed was replaced by blank feed. Day 22–24 are measured concentrations after administration of blank feed, hence representing the depletion of CIT. Figures S11–S20: histology of kidney, liver, duodenum or jejunum from pigs, broiler chickens and laying hens (20×; 40×, HE stain).
