# Supplementary material for: A Study of Carry-Over and Histopathological Effects after Chronic Dietary Intake of Citrinin in Pigs, Broiler Chickens and Laying Hens
Source: Toxins (Basel). 2020 Nov 16;12(11):719. doi: 10.3390/toxins12110719 (PMC7697729; doi:10.3390/toxins12110719)
Supplement: Supplementary file 1 [file toxins-12-00719-s001.pdf]

Supplementary Materials: A Study of Carry-Over and Histopathological Effects after Chronic Dietary Intake of Citrinin in Pigs, Broiler Chickens and Laying Hens

Celine Meerpoel, Arnau Vidal, Emmanuel K. Tangni, Bart Huybrechts, Liesbeth Couck, Riet De Rycke, Lobke De Bels, Sarah De Saeger, Wim Van den Broeck,, Mathias Devreese and Siska Croubels

Table S1. Validation parameters for citrinin (CIT) and dihydrocitrinone (HO-CIT) in edible tissue matrices for linearity (correlation coeffiecient R^2 and goodness-of-fit g), limit of detection (LOD), limit of quantification (LOQ).

**Animal**			**CIT**					**HO-CIT**	
	**Matrix**	**R^2**	*g*	**LOD (µg/kg)**	**LOQ (µg/kg)**	R^2	*g*	LOD (µg/kg)	LOQ (µg/kg)
**PIG**	**Muscle**	0.998	5.6	0.02	0.1	0.996	8.9	0.32	1
	**Liver**	0.998	4.8	0.03	0.5	0.993	10.2	0.04	0.5
	**Kidney**	0.999	3.0	0.03	0.5	0.999	7.2	1	5
	**Skin and fat**	0.997	21.3	0.1	0.5	0.988	23.2	0.5	1
**CHICKEN**	**Muscle**	0.999	5.1	0.02	0.1	0.999	15.8	0.32	1
	**Liver**	0.995	17.7	2.5	5	0.999	3.8	4.5	8
	**Kidney**	0.999	7.3	0.5	1	0.999	15.9	2.5	5
	**Skin and fat**	0.999	11.5	0,1	0.5	0.998	4.7	2.5	5
	**Egg yolk**	0.999	6.5	0.09	0.5	0.999	4.2	0.3	1
	**Egg white**	0.999	5.7	0.05	0.1	0.996	8.1	1	5

Table S2. Validation results for citrinin (CIT) and dihydrocitrinone (HO-CIT) for apparent recovery (R_{app}), within-day (RSD_r, n = 6) and between-day precision for muscle tissue (RSD_R, n = 3x3) at 3 concentration levels for pigs. RSD_R was not calculated for other matrices, as a shortened one-day validation was applied.

	CIT				HO-CIT			
**Matrix**	**Concentration (µg/kg)**	**R_{app} (%)**	**RSD_r (%)**	**RSD_R (%)**	**Concentration (µg/kg)**	R_{app} (%)	RSD_r (%)	RSD_R (%)
**Muscle**	0.1	108	15	21	1	97	5	31
	1	94	16	18	10	106	4	21
	50	104	9	11	50	101	14	24
**Liver**	0.5	101.7	5	-	1	105	30	-
	10	85	8	-	10	84	9	-
	200	109	4	-	50	98	4	-
**Kidney**	0.5	98	3	-	5	79	28	-
	10	86	5	-	10	99	5	-
	200	109	3	-	200	102	10	-
**Skin and fat**	5	103	24	-	1	95	4	-
	10	70	2	-	10	98	9	-
	100	82	37	-	50	76	5	-

Table S3. Validation results for citrinin (CIT) and dihydrocitrinone (HO-CIT) for apparent recovery (R_{app}), within-day (RSD_r, n = 6) precision at 3 concentration levels for chickens.

	CIT			HO-CIT		
**Matrix**	**Concentration (µg/kg)**	**R_{app} (%)**	**RSD_r (%)**	Concentration (µg/kg)	R_{app} (%)	RSD_r (%)
**Muscle**	0.5	98	10	5	122	19
	5	107	2	10	119	3
	50	107	7	50	110	1
**Liver**	5	88	15	10	92	17
	50	109	12	50	102	7
	200	99	5	100	99	7
**Kidney**	1	87	26	5	86	19
	10	106	9	10	84	27
	200	101	6	50	78	18
**Skin and fat**	0,5	101	11	5	73	16
	20	94	5	20	92	29
	100	97	7	100	104	5
**Egg yolk**	1	103	9	5	76	16
	10	113	5	10	93	10
	20	105	3	20	107	9
**Egg white**	1	105	2	1	99	0.1
	10	112	2	10	101	7
	20	107	6	20	109	11

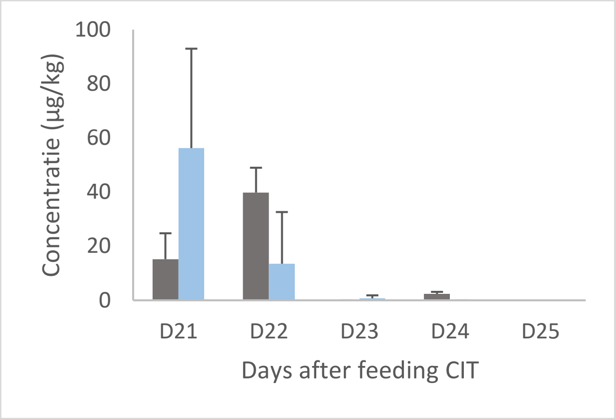

Figure S1. Citrinin (CIT) and dihydrocitrinone (HO-CIT) concentrations measured in kidney tissue from pigs after euthanasia. At day 21, the CIT contaminated feed was replaced by blank feed. Day 22-25 are measured concentrations after administration of blank feed, hence representing the depletion of CIT and HO-CIT.

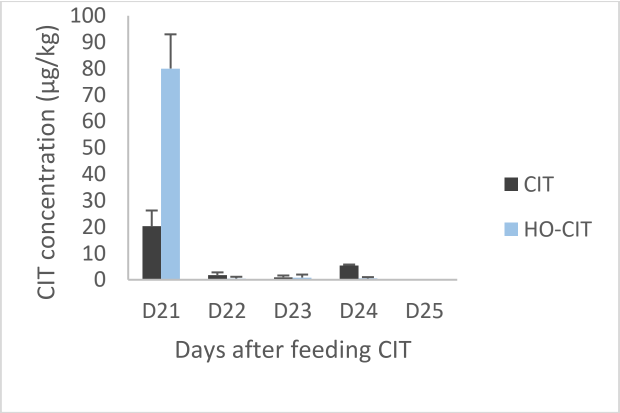

Figure S2. Citrinin (CIT) and dihydrocitrinone (HO-CIT) concentrations measured in liver tissue from pigs after euthanasia. At day 21, the CIT contaminated feed was replaced by blank feed. Day 22-25 are measured concentrations after administration of blank feed, hence representing the depletion of CIT and HO-CIT.

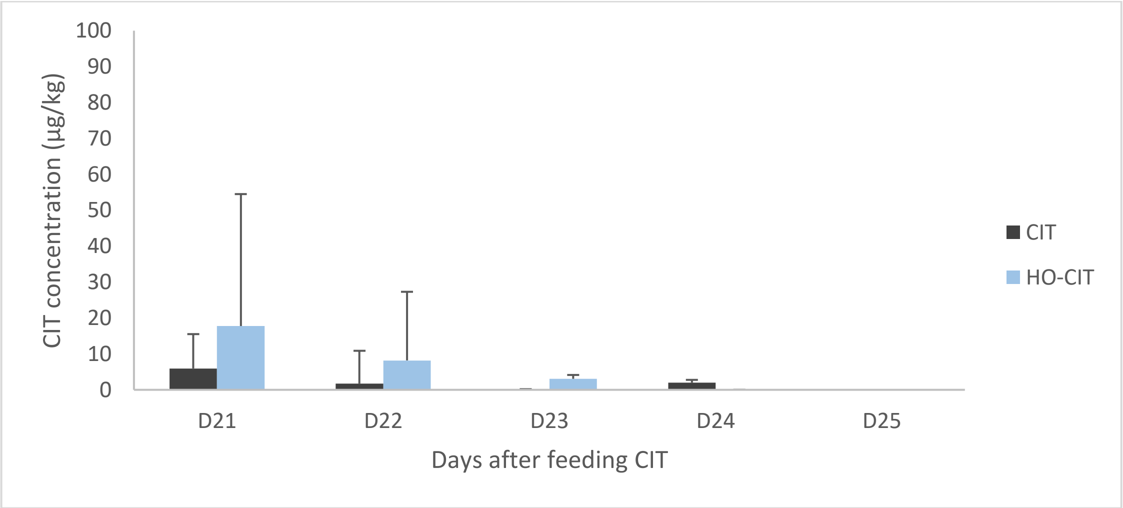

Figure S3. Citrinin (CIT) and dihydrocitrinone (HO-CIT) concentrations measured in skin and fat tissue from pigs after euthanasia. At day 21, the CIT contaminated feed was replaced by blank feed. Day 22-25 are measured concentrations after administration of blank feed, hence representing the depletion of CIT and HO-CIT.

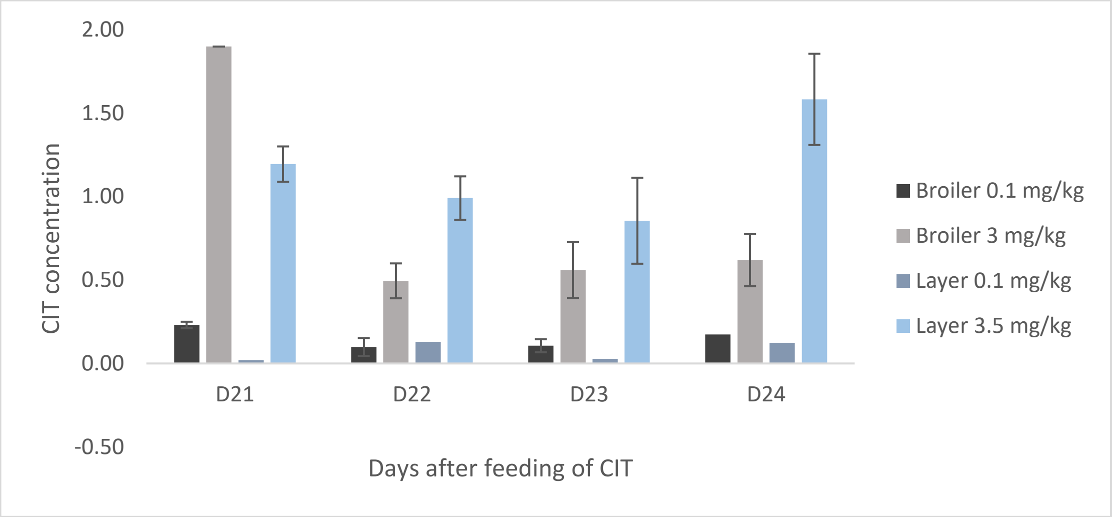

Figure S4. Citrinin (CIT) concentrations measured in kidney tissue from broilers chickens and layer hens after euthanasia. At day 21, the CIT contaminated feed was replaced by blank feed. Day 22-24 are measured concentrations after administration of blank feed, hence representing the depletion of CIT.

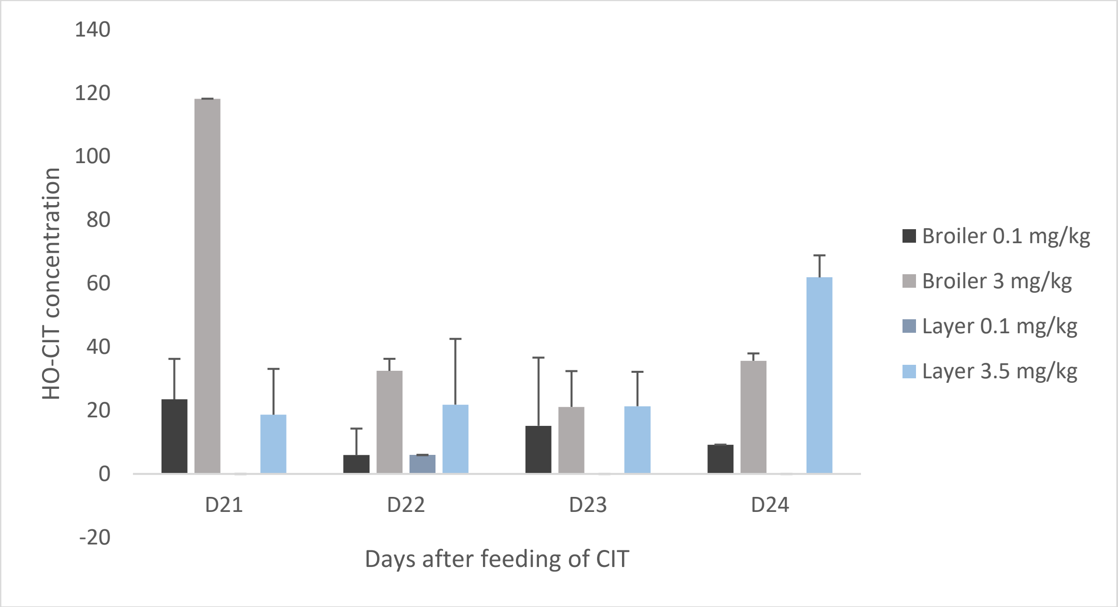

Figure S5. Dihydrocitrinone (HO-CIT) concentrations measured in kidney tissue from broilers chickens and layer hens after euthanasia. At day 21, the CIT contaminated feed was replaced by blank feed. Day 22-24 are measured concentrations after administration of blank feed, hence representing the depletion of HO-CIT.

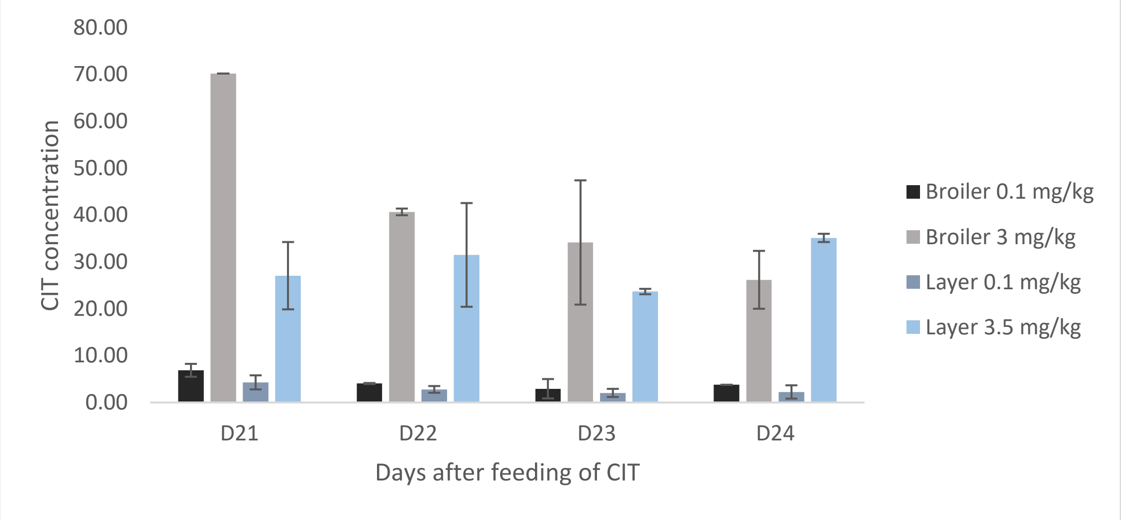

Figure S6. Citrinin (CIT) concentrations measured in liver tissue from broilers chickens and layer hens after euthanasia. At day 21, the CIT contaminated feed was replaced by blank feed. Day 22-24 are measured concentrations after administration of blank feed, hence representing the depletion of CIT.

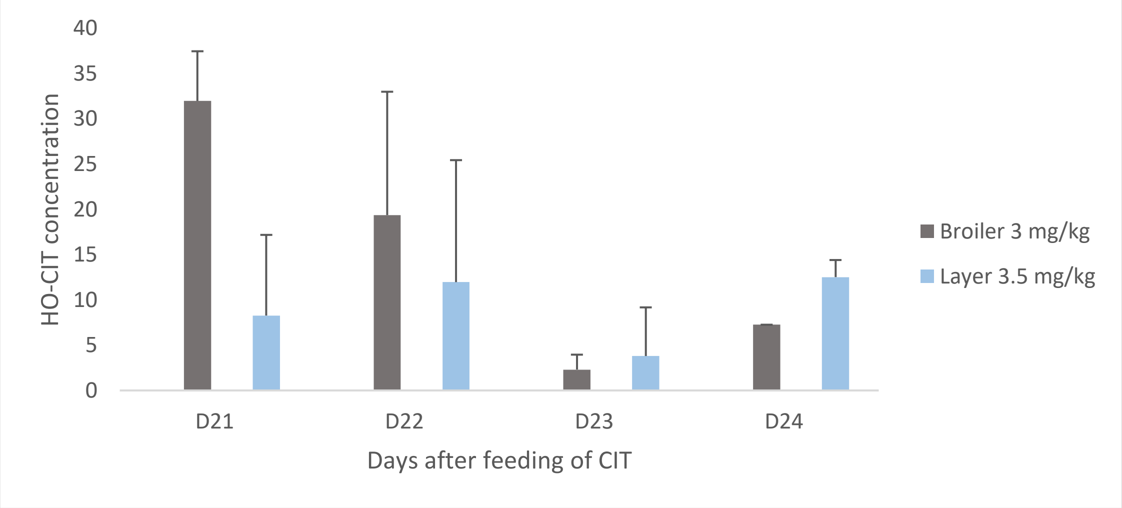

Figure S7. Dihydrocitrinone (HO-CIT) concentrations measured in liver from broilers chickens and layer hens after euthanasia. At day 21, the CIT contaminated feed was replaced by blank feed. Day 22-24 are measured concentrations after administration of blank feed, hence representing the depletion of HO-CIT.

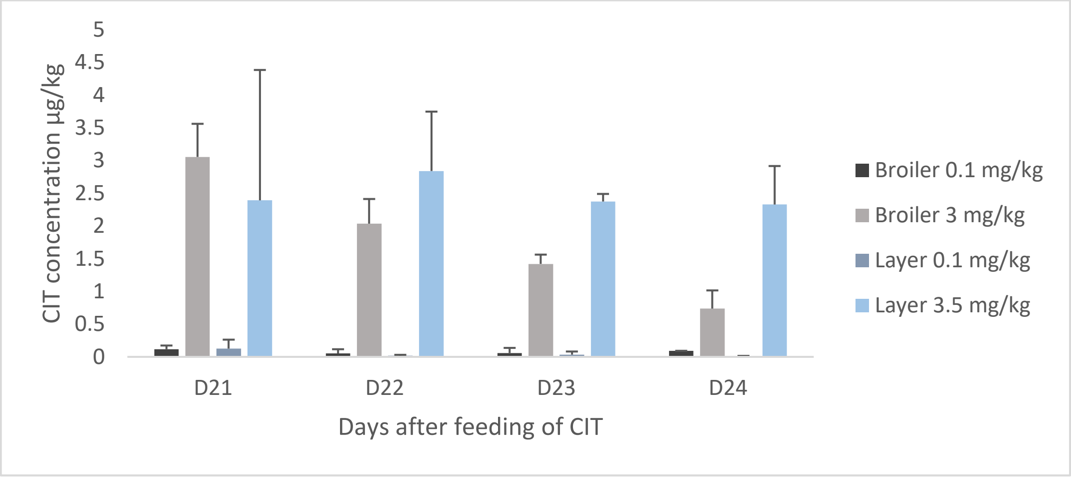

Figure S8. Citrinin (CIT) concentrations measured in muscle tissue from broilers chickens and layer hens after euthanasia. At day 21, the CIT contaminated feed was replaced by blank feed. Day 22-24 are measured concentrations after administration of blank feed, hence representing the depletion of CIT.

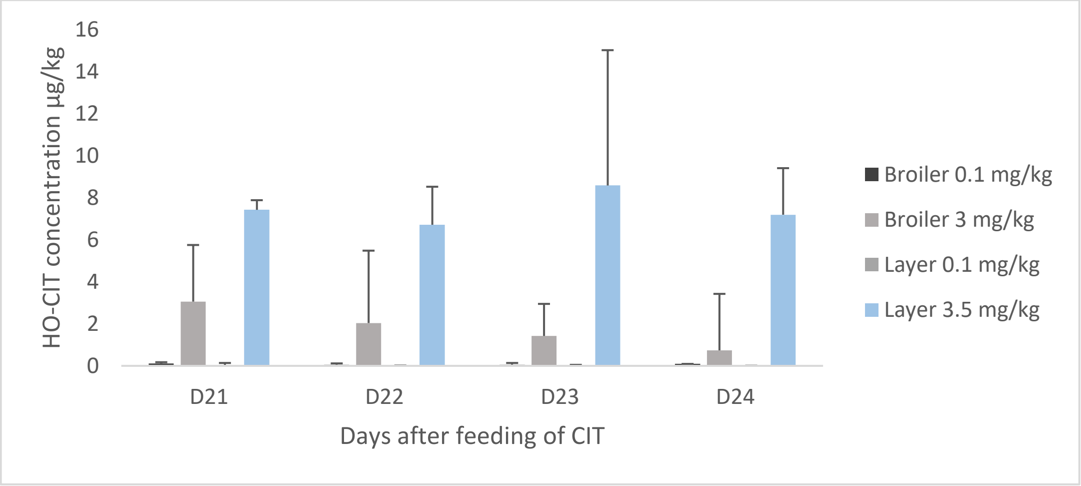

Figure S9. Dihydrocitrinone (HO-CIT) concentrations measured in muscle tissue from broilers chickens and layer hens after euthanasia. At day 21, the CIT contaminated feed was replaced by blank feed. Day 22-24 are measured concentrations after administration of blank feed, hence representing the depletion of HO-CIT.

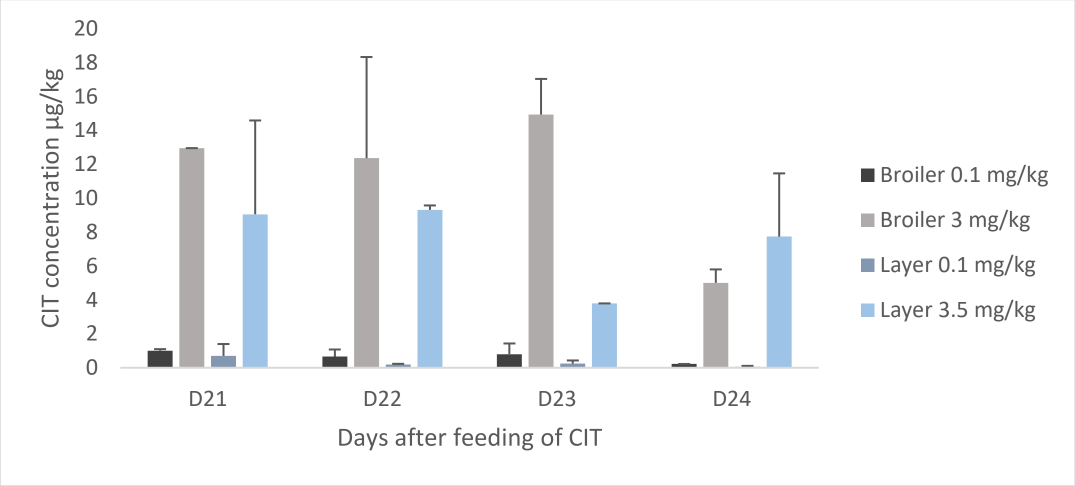

Figure S10. Citrinin (CIT) concentrations measured in skin and fat tissue from broilers chickens and layer hens after euthanasia. At day 21, the CIT contaminated feed was replaced by blank feed. Day 22-24 are measured concentrations after administration of blank feed, hence representing the depletion of CIT.

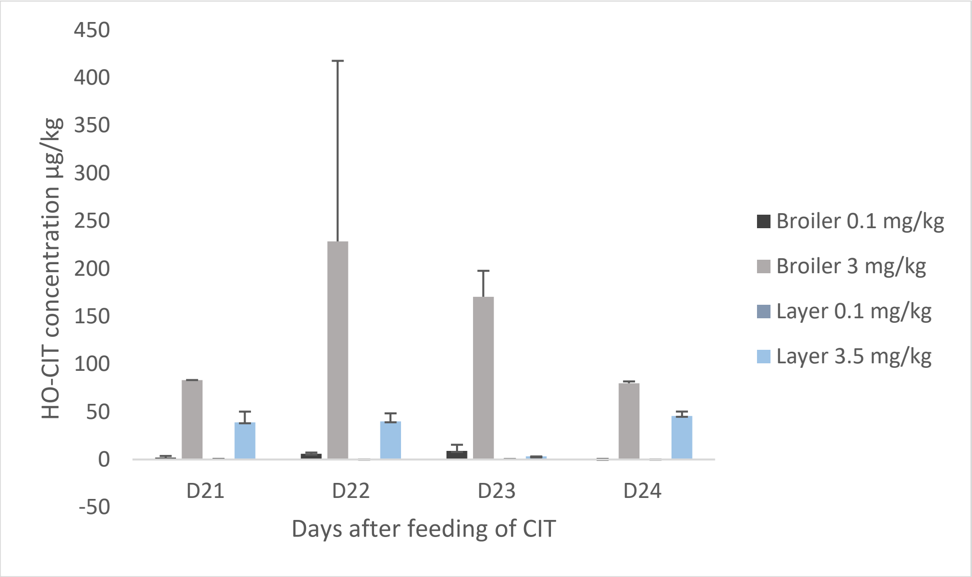

Figure S11. Dihydrocitrinone (HO-CIT) concentrations measured in skin and fat tissue from broilers chickens and layer hens after euthanasia. At day 21, the CIT contaminated feed was replaced by blank feed. Day 22-24 are measured concentrations after administration of blank feed, hence representing the depletion of HO-CIT.

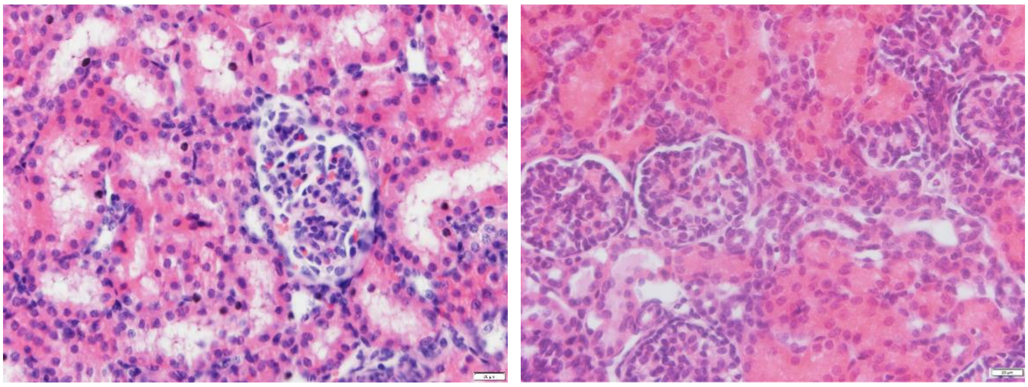

Figure S12. Histology of pig kidney cortex (40x, HE stain). Left: control pig; right: pig received 1 mg/kg feed.

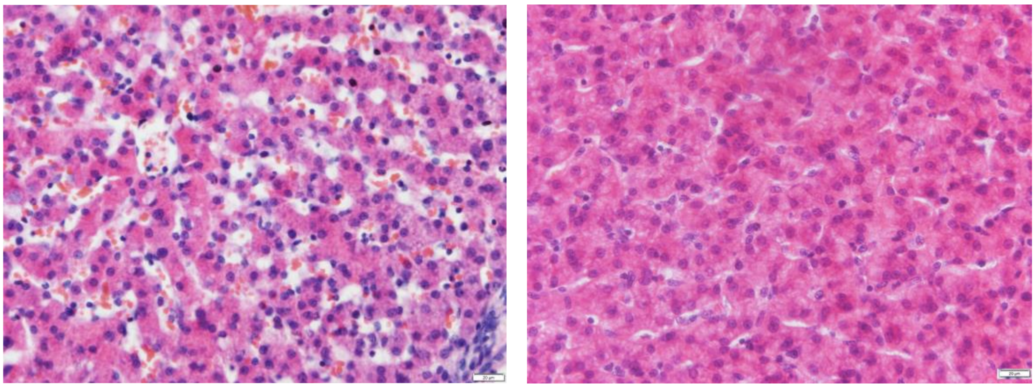

Figure S13. Histology of pig liver (40x, HE stain). Left: control pig; right: pig received 1 mg/kg feed.

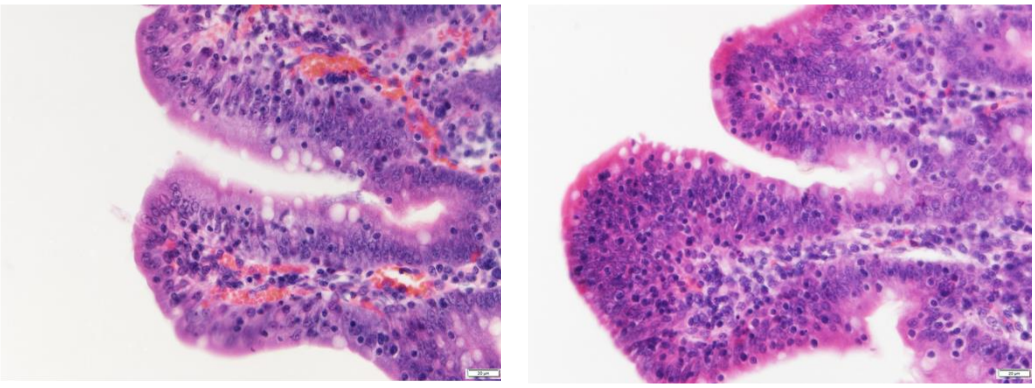

Figure S14. Histology of pig duodenum (40x, HE stain). Left: control pig; right: pig received 1 mg/kg feed.

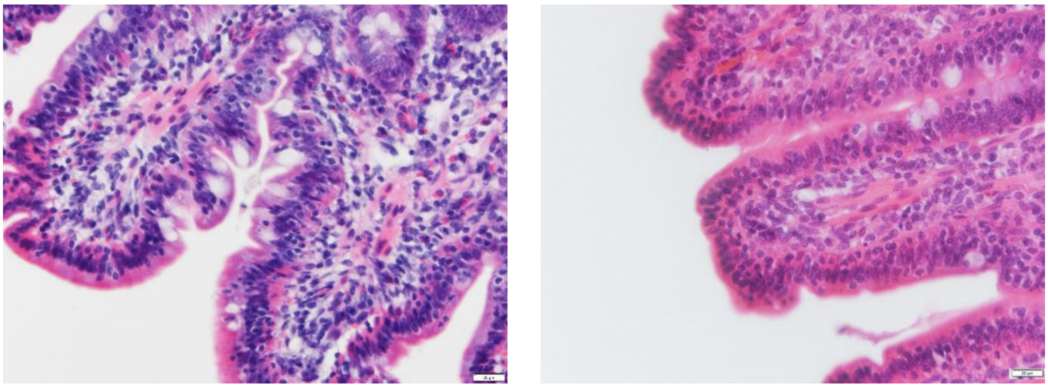

Figure S15. Histology of pig jejunum (40x, HE stain). Left: control pig; right: pig received 1 mg/kg feed.

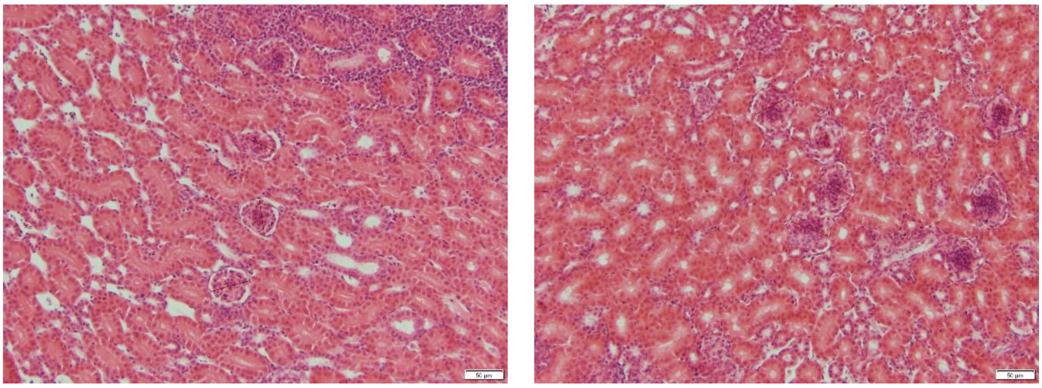

Figure S16. Histology of broiler kidney cortex (20x, HE stain). Left: control broiler; right: broiler received 3 mg/kg feed.

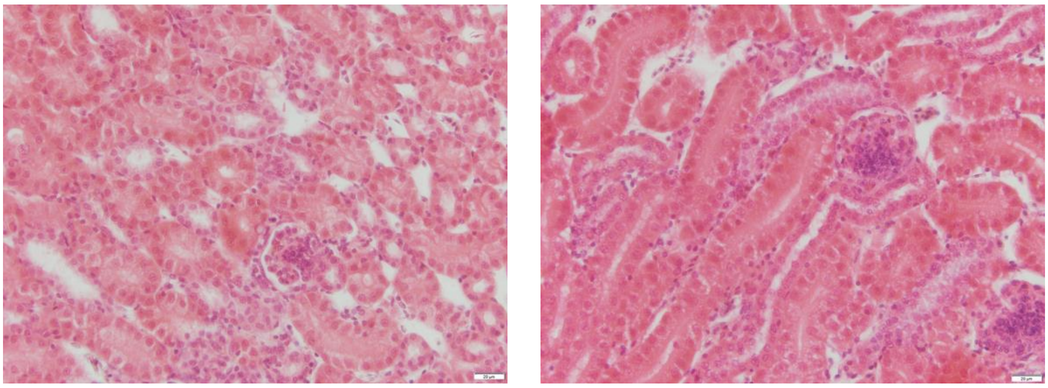

Figure S17. Histology of layer kidney cortex (40x, HE stain). Left: control layer; right: layer received 3.5 mg/kg feed.

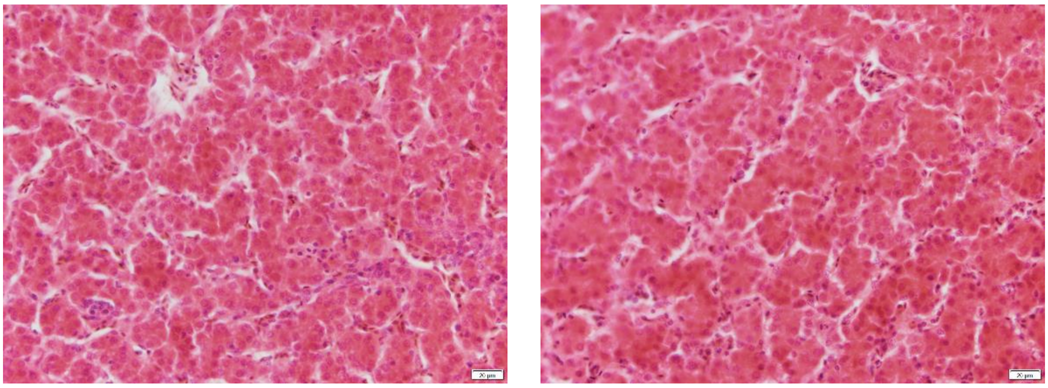

Figure S18. Histology of broiler liver (40x, HE stain). Left: control broiler; right: broiler received 3 mg/kg feed.

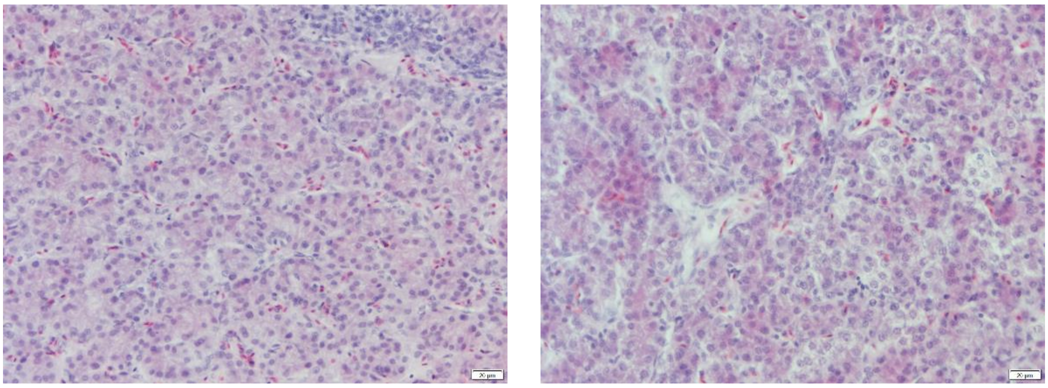

Figure S19. Histology of layer liver (40x, HE stain). Left: control layer; right: layer received 3.5 mg/kg feed.

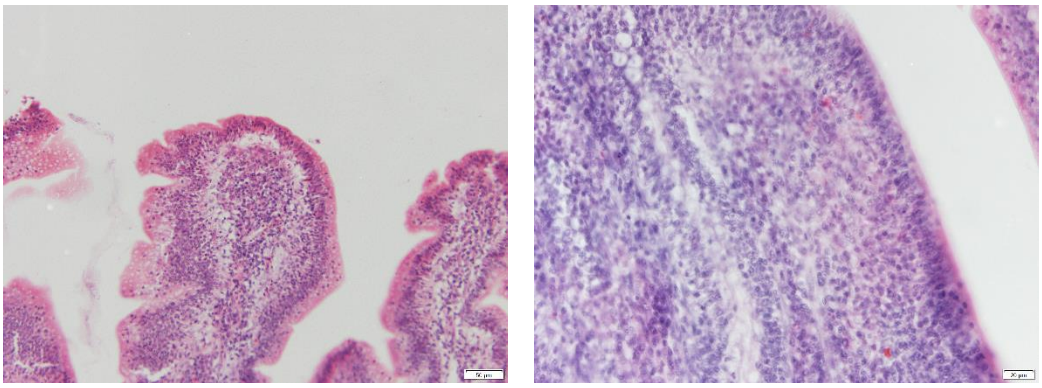

Figure S20. Histology of broiler jejunum (20x;40x, HE stain). Left: control broiler; right: broiler received 3 mg/kg feed. Jejunum of layer and duodenum of both species is not available due to technical problems.
